# Decreased TSPAN14 Expression Contributes to NSCLC Progression

**DOI:** 10.3390/life12091291

**Published:** 2022-08-23

**Authors:** Mirna Jovanović, Tijana Stanković, Sonja Stojković Burić, Jasna Banković, Jelena Dinić, Mila Ljujić, Milica Pešić, Miodrag Dragoj

**Affiliations:** 1 Department of Neurobiology, Institute for Biological Research “Siniša Stanković”—National Institute of the Republic of Serbia, University of Belgrade, Despota Stefana 142, 11060 Belgrade, Serbia; 2 Institute of Molecular Genetics and Genetic Engineering, University of Belgrade, Vojvode Stepe 444a, 11042 Belgrade, Serbia

**Keywords:** Tspan14, lung cancer, NSCLC, metastasis

## Abstract

Tspan14 is a transmembrane protein of the tetraspanin (Tspan) protein family. Different members of the Tspan family can promote or suppress tumor progression. The exact role of Tspan14 in tumor cells is unknown. Earlier, mutational inactivation of the TSPAN14 gene has been proposed to coincide with a low survival rate in NSCLC patients. This study aimed to investigate the correlation of TSPAN14 lack of function with clinicopathological features of NSCLC patients, and to elucidate the role TSPAN14 might have in NSCLC progression. TSPAN14 expression was lower in tumor cells than non-tumor cells in NSCLC patients’ samples. The decreased gene expression was correlated with a low survival rate of patients and was more frequent in patients with aggressive, invasive tumor types. Additionally, the role of decreased TSPAN14 expression in the metastatic potential of cancer cells was confirmed in NSCLC cell lines. The highly invasive NSCLC cell line (NCI-H661) had the lowest TSPAN14 gene and protein expression, whereas the NSCLC cell line with the highest TSPAN14 expression (NCI-H460) had no significant metastatic potential. Finally, silencing of TSPAN14 in these non-metastatic cancer cells caused an increased expression of matrix-degrading enzymes MMP-2 and MMP-9, followed by an elevated capacity of cancer cells to degrade gelatin. The results of this study propose TSPAN14 expression as an indicator of NSCLC metastatic potential and progression.

## 1. Introduction

In 2020, there were over 19.3 million recorded new cancer cases; by 2040, the estimates indicate there will be a 47% rise relative to the current state [1]. Cancer progression leading to a fatal outcome mainly depends on the ability of cancer cells to invade and migrate from the primary location and form tumor masses at distant sites [2]. The initial event in forming a metastatic cancer cell is obtaining the ability to detach from the tumor, reorganize the surrounding matrix and move toward blood vessels.

Lung cancer is the second most-diagnosed cancer in the world, with over 2 million newly diagnosed cases per year [1], and remains the leading cause of cancer-caused death worldwide [1,3,4]. Lung cancer is primarily classified as small-cell lung carcinoma (SCLC) and non-small cell lung carcinoma (NSCLC), among which NSCLC makes up the large majority of diagnosed cases. The socioeconomic burden of NSCLC cancer incidence would decelerate significantly with improvements in prevention strategies and a patient-specific approach to therapy. The identification of new molecular mechanisms contributing to the progression of the disease, factors indicating a risk of the metastatic cascade, and biomarkers for targeted therapy, would make an appreciable contribution to the efforts of global lung cancer control.

Tetraspanins (Tspans), a family of transmembrane proteins, are of critical significance in the regulation of cancer invasion and metastasis through the control of cell migration and cancer-endothelial cell interactions [5,6]. The physiological role of these proteins is the regulation of cell proliferation, migration, differentiation, and development. Tspans consist of four membrane-spanning domains, a large and a small extracellular domain, three short intracellular domains, and cytoplasmic N- and C-termini [7,8]. The family of 33 members has a cell-specific expression profile, some of them being ubiquitously expressed, while others are highly determined [5]. Apart from the cell surface, they are also present in extra- and intracellular vesicles’ lipid bilayers, thus contributing to cell trafficking and cell–cell signalization [9,10,11]. The Tspan protein family forms Tspan-enriched membrane areas (TEMs) through interaction with other transmembrane and intracellular proteins, organizing adhesion, adaptor, and signaling molecules into microdomains [10,12]. The main mechanism of Tspans affecting migration and metastasis in cancer cells is through interaction with integrins, the mediators between the cell cytoskeleton and extracellular matrix (ECM). However, whether Tspans will promote or suppress the invasiveness in cancer cells is context-dependent owing to different tumor types, Tspan proteins, and TEM scaffolds [5].

Our earlier study showed that mutational inactivation in the *TSPAN14* gene could play a role in NSCLC promotion [13]. Tspan14 belongs to a subfamily of six related proteins—the TspanC8 group (the “C8“ refers to the 8 cysteine residues in the large extracellular domain) [8,14]. TspanC8 proteins have been proposed as regulators of a Notch-activating A Disintegrin and Metalloprotease 10 (ADAM10) in *Drosophila* and mammalian platelets [14,15]. Notch acts as both a tumor promoter and suppressor, depending on the tumor type. For example, overexpressed Notch-1 promotes epithelial-mesenchymal transition (EMT), and consequently, invasion and migration of tumor cells in ovarian [16], breast [17], and lung cancers [18], while in NSCLC, downregulated Notch-3 plays a tumor-suppressive role [19]. To the best of our knowledge, the functional significance of *TSPAN14* expression levels in NSCLC cancer cells was not established previously.

In this study, we analyzed the expression of *TSPAN14* in NSCLC patient samples. The decreased *TSPAN14* expression incidence was related to the major histopathological parameters and the effects on patients’ survival rates. Furthermore, we determined *TSPAN14* expression profiles in NSCLC cell lines and examined their migratory and invasive potential. Using these analyses, we aimed to address whether the *TSPAN14* gene has a role in regulating the migration and invasion of NSCLC cells and serves as a prognostic factor in NSCLC.

## 2. Materials and Methods

### 2.1. Tissue Samples

The study included samples of 40 NSCLC patients acquired during tumor surgical resection at the Clinic for Thoracic Surgery, University Clinical Centre of Serbia (Belgrade, Serbia). The primary tumor, as well as the surrounding non-tumor lung tissue samples, were immediately frozen in liquid nitrogen and kept there until further analysis. Histopathological examination at the Department of Thoracic Pathology, Service of Pathology, University Clinical Centre of Serbia (Belgrade, Serbia) confirmed the diagnosis of NSCLC and classified the tumor by histological subtype, grade stage, pleural and lymph node invasion. The tissue samples’ clinicopathological parameters are described in Table 1. The patients received neither radiotherapy nor chemotherapy before surgery.

### 2.2. Cell Culture

The A549, HaCaT, NCI-H661, and NCI-H460 cell lines were obtained from the American Type of Culture Collection (ATCC, Manassas, VA, USA). All cells were maintained at 37 °C in humidified 5% CO_2_ atmosphere in RPMI-1640 medium, supplemented with 10% fetal bovine serum (FBS), 2 mM L-glutamine, 4.5 g/L glucose, 10,000 U/mL penicillin, 10 mg/mL streptomycin and 25 μg/mL amphotericin B solution. The cells were sub-cultured after reaching the 80% confluence using 0.25% trypsin/EDTA.

### 2.3. Protein Expression Analysis

Flow cytometry was used for the analysis of Tspan14 protein expression. The cells were trypsinized, counted, and divided into groups (200,000 cells per sample), washed in phosphate buffer solution (PBS), and fixed in 4% paraformaldehyde (PFA) for 10 min at room temperature. The cells were then permeabilized with ice-cold 90% methanol for 30 min at 4 °C. After washing in PBS, the samples were blocked with 0.5% bovine serum albumin solution in PBS (BSA) for 1 h and incubated overnight at 4 °C with polyclonal anti-Tspan14 antibody (PA5-37979, Thermo Fisher Scientific, Waltham, MA, USA) diluted 1:50 in 0.5% BSA or kept overnight only with 0.5% BSA (for determination of relative Tspan14 expression). The cells were washed in PBS and incubated for 30 min at room temperature with fluorescent anti-goat IgG (H + L) secondary antibody (AlexaFluor^®^488 Conjugated, #4412, Cell Signaling Technology, Danvers, MA, USA) diluted 1:1000 in 0.5% BSA. The cells were then washed and resuspended in 1-mL PBS. The mean fluorescence intensity (MFI) was measured in the FL1 channel on a CyFlow Space flow cytometer (Partec, Münster, Germany). A minimum of 10,000 events was recorded per sample. The collected data were analyzed using Summit 4.3 software (Dako Colorado Inc., Fort Collins, CO, USA). To determine relative Tspan14 expression, MFI obtained for each cell line after labeling with polyclonal anti-Tspan14 antibody and fluorescent anti-goat IgG (H + L) secondary antibody was divided by MFI of particular cell line labeled only with fluorescent anti-goat IgG (H + L) secondary antibody.

### 2.4. siRNA Transfection of NCI-H460 Cell Line

NCI-H460 cells were seeded at a density of 200,000 cells per well on a 6-well plate and grown for 24 h, or until reaching 80% confluence, in an antibiotic-free RPMI medium with 10% FBS and 2 mM L-glutamine. The cells were transfected with a final concentration of 50 nmol predesigned *TSPAN14*-specific siRNA (5′-GGAUUCAGCUGAAGAGCAATT-3′ and 5′-UUGCUCUUCAGCUGAAUCCTG-3′) or the same concentration of scrambled control siRNA using Lipofectamine 3000 reagent (Invitrogen Life Technologies, Carlsbad, CA, USA) according to manufacturer’s protocol. After 24 h, the transfection complex containing medium was removed, and the cells were further used for immunohistochemistry, RNA extraction, or invasion and migration assays.

### 2.5. Immunostaining and Fluorescence Microscopy

Transfected NCI-H460 cells were seeded (25,000 cells/chamber) in 8-well chamber slides (Nunc, Nalgene, Roskilde, Denmark) in 500 µL of RPMI medium and grown overnight. The cells were washed in PBS, fixed in 4% PFA for 15 min at 4 °C, and blocked in 0.5% BSA for 1 h. Anti-Tspan14 antibody (PA5-37979, Thermo Fisher Scientific, USA) was applied at 1:50 dilution in 0.5% BSA, and the cells were incubated overnight at 4 °C. After washing in PBS, fluorescent anti-goat IgG (H + L) secondary antibody (AlexaFluor^®^488 Conjugated, #4412, Cell Signaling Technology, USA) was applied at 1:1000 dilution in 0.5% BSA for 1 h at room temperature. To mark the nuclei, the cells were co-stained with Hoechst 33342 (Sigma-Aldrich Chemie GmbH, Schnelldorf, Germany) and then mounted in Mowiol. The cells were imaged on ZOE Fluorescent Cell Imager (Bio-Rad Laboratories, Hercules, CA, USA) using a 20× objective.

To quantify the Tspan14 expression, the fluorescence intensity of anti-Tspan14-labeled cells in captured images was analyzed using ImageJ software (U.S. National Institutes of Health, Hercules, CA, USA). The corrected total cell fluorescence (CTCF) corresponding to the Tspan14 signal was calculated using the following formula: CTCF = Integrated density − (Area × Mean fluorescence of background readings). To measure the background, three areas not containing cells were selected for each image, and mean fluorescence was determined. CTCF in each analyzed area was divided by the number of cells in the area. Five independent fields were analyzed per each image and the results are presented as the CTCF per cell.

### 2.6. RNA Extraction and Reverse Transcription

Total RNA was isolated from tumor and corresponding normal tissue samples of all 40 NSCLC patients, as well as from the cell lines. TRIzol^®^ Reagent (Invitrogen Life Technologies, Waltham, MA, USA) was used to isolate the total RNA according to the manufacturer’s instructions. The RNA concentrations were determined by spectrophotometry, and quality was verified by electrophoresis on 1.2% agarose. A reverse transcription reaction was performed using a High-Capacity cDNA Reverse Transcription Kit (Applied Biosystems, Waltham, MA, USA) and 2 μg of total RNA, following the manufacturer’s protocol.

### 2.7. Quantitative Real-Time PCR

Quantitative real-time PCR (qRT-PCR) was used to evaluate mRNA levels of *TSPAN14*, *MMP2*, and *MMP9*. Gene expression levels of *TSPAN14* and *HPRT* (as a reference gene for the normalization of target mRNA expression), were detected using Applied Biosystems™ TaqMan™ Gene Expression Assay (ThermoFisher Scientific, Waltham, MA, USA). Primers and probes specific to *TSPAN14* and *HPRT* were obtained from Applied Biosystems as following Assay-on-Demand Gene Expression Products: *TSPAN14* (Hs00229502_m1) and *HPRT1* (Hs01003267_m1). The expression analysis of *MMP2*, *MMP9*, and *ACTB*, as a reference gene, was performed with Maxima SYBR Green/ROX qPCR Master Mix (ThermoFisher Scientific, USA). The primer sequences used in SYBR Green/ROX assay were as follows: 5′-CCG TCG CCC ATC ATC AAG TT-3′ and 5′-CTG TCT GGG GCA GTC CAA AG-3′ for *MMP2*, 5′-GGG ACG CAG ACA TCG TCA TC-3′ and 5′-TCG TCA TCG TCG AAA TGG GC-3′ for *MMP9*, and 5-TGG ACA TCC GCA AAG ACC TGT AC-3 and 5-TCA GGA GGA GCA ATG ATC TTG A-3 for the *ACTB*. qRT-PCR was performed using the QuantStudio 3 Real-Time PCR system (ThermoFisher Scientific, USA). Each sample was evaluated in triplicate and relative gene expression levels were analyzed by the 2^−ΔΔCt^ method [20].

### 2.8. Gelatin Degradation Assay

A gelatin degradation assay was used to test the ability of cells to degrade the ECM. The model used was gelatin conjugated to a fluorescent green dye (Gelatin From Pig Skin, Oregon Green^®^ 488 Conjugate, Life Technologies, Carlsbad, CA, USA). Coverslips were coated with AlexaFluor^®^488 labeled gelatin, placed in a 6-well plate and 50,000 cells/well were seeded on the top of the coated coverslips. After 24-h incubation, the cells were fixed with 4% PFA, washed in PBS, and stained with Hoechst33342 (Sigma-Aldrich Chemie GmbH, Berlin, Germany) and ActinRed^®^555 (Life Technologies, San Diego, CA, USA) for 1 h at room temperature. The number of cells and degraded areas was visualized at 20× magnification under a Zeiss Axiovert inverted fluorescence microscope (Carl Zeiss Foundation, Stuttgart, Germany) equipped with AxioVision 4.8 Software. The volume of the dark areas caused by the degradation of gelatin was measured using ImageJ software and normalized to the number of cells. All experiments were performed at least thrice.

### 2.9. Invasion Assay

Invasion assay was used to assess the ability of the cell to pass through a porous Matrigel^®^ matrix-coated membrane (Transwell inserts, pore size 8 μm; diameter, 6.4 mm; BD Biosciences, Franklin Lakes, NJ, USA). Transwell inserts with a porous membrane were placed in a cell culture 24-well plate. The upper surface of the membrane was coated with a thin layer of Matrigel^®^ matrix, diluted 1:15 in a culture medium without FBS (500 ng/mL), and the gel polymerized for 1 h at 37 °C. 200,000 cells in serum-free medium per insert were seeded. The lower chambers were filled with culture medium, supplemented with 10% FBS as a chemoattractant. Control of spontaneous cell invasion was included, using RPMI-1640 medium without 10% FBS in lower chambers. After 24-h incubation, non-migrated cells from the top membranes were removed, and the cells that migrated through the membranes were fixed in 4% PFA, stained with Hoechst 33342, and imaged under a Zeiss Axiovert inverted fluorescence microscope (Carl Zeiss Foundation, Germany) equipped with AxioVision 4.8 Software at 10× magnification. The average number of cells in 10 independent fields per membrane was analyzed. At least three independent experiments were performed. Results are presented as the percentage of cells that invaded through the matrix-coated membrane.

### 2.10. Statistics

Statistical analyses were performed using the statistical software R (R version 4.0.2, Copyright (C) 2020 The R Foundation for Statistical Computing) and GraphPad Prism 6.0 software. The data obtained from qRT-PCR analyses, flow cytometric analysis, gelatin degradation assay, and the invasive assay were analyzed using a Student *t*-test or Mann-Whitney test. Fisher exact test was used to examine the relationship between histopathological parameters (NSCLC subtype, histological grade, stage, lymph node invasion, and pleural invasion) and lowered *TSPAN14* gene expression. Survival analyses were performed using Kaplan and Meier product-limit method. The log-rank test was used to assess the significance of the difference between pairs of survival probabilities. The associations between overall survival, *TSPAN14* expression, and clinical histopathological parameters were assessed using a Cox proportional hazards model. The overall survival rate was calculated from day one after surgery to the last follow-up examination or death of the patient. Statistical differences were considered significant when the *p*-value was <0.05.

## 3. Results

### 3.1. Decreased TSPAN14 Gene Expression Is Associated with Pleural Invasion in Patients with NSCLC

**Figure 1:**
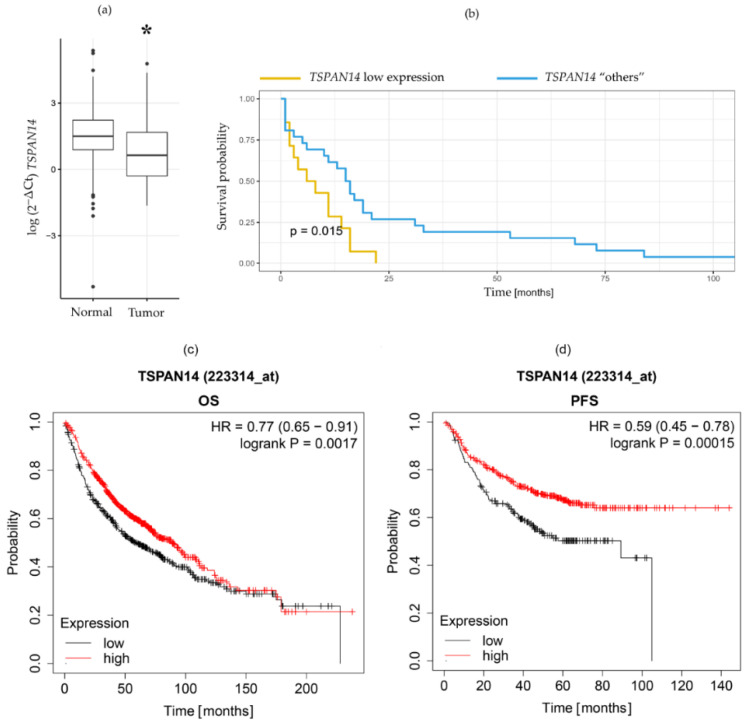
Decreased expression of *TSPAN14* in NSCLC patients is correlated with a lower survival rate. (**a**) *TSPAN14* gene expression in tumor tissue and the surrounding normal lung tissue in NSCLC patients. *TSPAN14* expression was normalized to *HPRT* as endogenous control. Normalized ΔCt values are presented in a logarithmic scale. *p* < 0.05 (*) indicate a statistically significant difference in *TSPAN14* expression in tumor samples compared to normal samples. (**b**) Kaplan-Meier survival curves show the NSCLC patients with *TSPAN14* decreased expression (“*TSPAN14* low expression”) and the NSCLC patients with higher *TSPAN14* expression (“*TSPAN14* others”). Low *TSPAN14* gene expression is indicated with a 3-fold decrease in the tumor compared to the matching normal tissue sample, and the differences were statistically significant (*p* < 0.05). (**c**) Kaplan-Meier plotter graph survival curves showing OS of NSCLC patients with *TSPAN14* low expression and the NSCLC patients with high *TSPAN14* expression (n = 1144); (**d**) Kaplan-Meier plotter graph showing PFS of NSCLC patients with *TSPAN14* low expression and the high *TSPAN14* expression (n = 591).

*TSPAN14* expression was analyzed by RT-qPCR in 40 NSCLC patients’ samples and paired with normal lung tissue. When compared to the normal lung tissue, tumor samples displayed significantly decreased *TSPAN14* expression (*p* = 0.0005 (Figure 1a). Namely, *TSPAN14* expression was lower in 14 of 40 tumor samples (35%, Table 1).

Furthermore, the significance of low *TSPAN14* expression was evaluated related to major histopathological parameters (NSCLC subtype, histological grade, stage, lymph node invasion, and pleural invasion). Statistical analyses revealed that lower *TSPAN14* expression was significantly associated with pleural invasion (*p* = 0.044, Table 1). In the group without pleural invasion, only 25.8% of patients had decreased *TSPAN14* expression (Table 1), whereas with pleural invasion, decreased expression of *TSPAN14* was more frequently present (66.6% of patients, Table 1).

### 3.2. Low TSPAN14 Gene Expression Could Be an Indicator of Poor NSCLC Patient Survival

Kaplan-Meier survival curves were generated to evaluate the influence of decreased *TSPAN14* expression on NSCLC patients’ survival. *TSPAN14* gene expression was considered low when the expression was decreased 3-fold in the tumor compared with the matching normal tissue sample and the differences were statistically significant (*p* < 0.05). Figure 1 shows the shorter survival of NSCLC patients with decreased *TSPAN14* expression. Specifically, the median survival time was 7 months for the patients with decreased *TSPAN14* expression, compared with 15.5 months for the other patients. Moreover, we used the log-rank test to determine the differences between the survival curves. Based on the log-rank test, the survival of the NSCLC patients with decreased *TSPAN14* expression was statistically different compared to the survival of the other patients (*p* = 0.015, Figure 1b). Using Cox regression, low *TSPAN14* expression was found to be significant for worse survival in univariable analysis (Figure S1, hazard ratio 2.36 (1.16–4.83), *p* = 0.02), and an independent predictor in multivariable analysis (Figure S1, hazard ratio 2.69 (1.01–7.17), *p* = 0.049). Furthermore, data available on the Kaplan–Meier plotter page were screened for the association of *TSPAN14* expression (mRNA gene chip) with overall survival (OS, Figure 1c) and progression-free survival (PFS, Figure 1d) of lung tumor patients [21]. The findings showed that low expression levels of *TSPAN14* were associated with poor OS (*p* = 0.0017) and PFS (*p* = 0.00015).

### 3.3. NSCLC Cell Lines with Low TSPAN14 Expression Have Increased Invasive Potential

**Figure 2:**
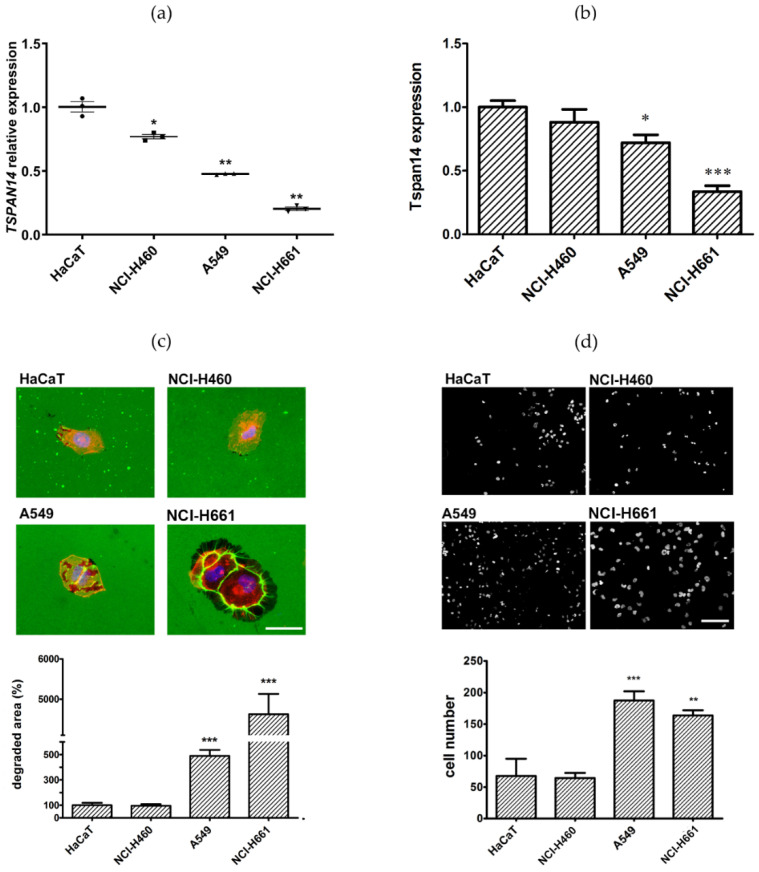
The expression of Tspan14 in normal and NSCLC cell lines and the cell lines’ potential to invade and migrate. (**a**) Quantitative real-time PCR analysis of *TSPAN14* gene expression, normalized to HPRT endogenous control, and (**b**) flow cytometry analysis of Tspan14 protein expression in permeabilized NCI-H460, A549, and NCI-H661 cancer cells, relative to normal human keratinocytes (HaCaT); (**c**) Representative images of gelatin degradation by HaCaT, NCI-H460, A549, and NCI-H661, attributed to histograms show a degraded gelatin area normalized to the total number of cells (relative degraded area). The scale bar in the figures marks a length of 50 μm. (**d**) Representative images of HaCaT, NCI-H460, A549, and NCI-H661 that migrated through the Matrigel^®^ matrix to the other side of the porous membrane; the histograms represent an average number of cells per field, in 10 independent fields per membrane, from three independent experiments. The scale bar in the pictures marks a length of 100 μm. All results are from at least three independent experiments. *p* < 0.05 (*), *p* < 0.01 (**), and *p* < 0.001 (***) indicate statistically significant differences between cancer cell lines and a normal cell line.

A normal cell line of human keratinocytes (HaCaT) and three NSCLC cell lines (NCI-H460, A549, and NCI-H661) were used to evaluate *TSPAN14* gene and protein expression. All three NSCLC cell lines had significantly decreased levels of the *TSPAN14* gene compared with the HaCaT cells (Figure 2a). NCI-H460 cells exhibit a 1.3-fold decreased expression (*p* = 0.047), A549 cells exhibited a 2.1-fold decreased expression (*p* = 0.013), and NCI-H661 cells exhibited a 5-fold decreased expression (*p* = 0.010) of the *TSPAN14* gene compared with the HaCaT cells.

The expression of Tspan14 protein in HaCaT and NSCLC cell lines was analyzed by flow cytometry (Figure 2b). It was found that A549 and NCI-H661 have significantly lower Tspan14 expression compared to HaCaT (1.4-fold, *p* = 0.027, and 3-fold, *p* = 0.0017, respectively), while statistically significant differences were not observed between NCI-H460 and HaCaT cells.

Next, a gelatin degradation assay was performed to compare the capacity of cell lines to degrade the extracellular matrix. In Figure 2c, it can be perceived that HaCaT and NCI-H460 cells have poor gelatin degradation ability. In contrast, A549 and NCI-H661 have a significantly higher ability to degrade gelatin. Specifically, A549 cells degraded gelatin with 4.9-fold higher efficacy than HaCaT cells, while NCI-H661 showed 46 times higher performance compared to HaCaT cells.

Finally, an invasion assay was used to test the ability of cell lines to degrade the matrix and pass through the membrane (Figure 2d). Analogous to gelatin degradation assay results, the invasion assay showed no difference between HaCaT and NCI-H460 cells, whereas A549 and NCI-H661 cells were notably more potent in passing through the membrane. Compared to HaCaT, A549 and NCI-H661 cells invaded through the membrane to a significantly higher extent (2.75-fold (*p* = 0.0006) and 2.4-fold (*p* = 0.0018), respectively).

### 3.4. Silenced-TSPAN14 NSCLC Cells Demonstrate the Increased Invasive Potential

**Figure 3:**
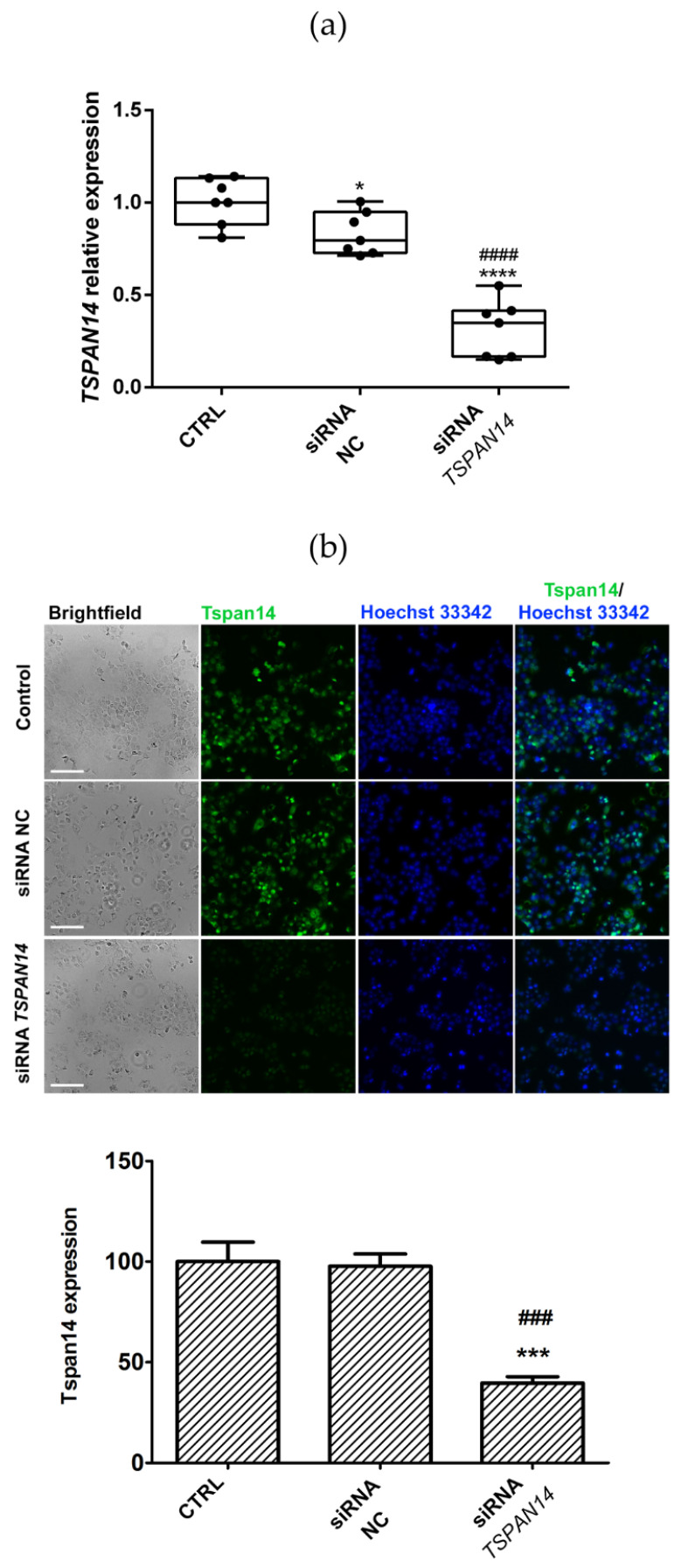
Transfection with siRNA *TSPAN14* for 24 h reduces *TSPAN14* expression in NCI-460. (**a**) Quantitative real-time PCR analysis of the *TSPAN14* expression normalized to *HPRT* as endogenous control, in non-transfected NCI-H460 cells (control), the cells transfected with non-coding siRNA (siRNA NC), and siRNA *TSPAN14* transfected NCI-H460 cells; (**b**) Representative micrographics of specific fluorescent labeling of Tspan14 protein (green) with the ascribed histogram quantification in control, siRNA NC and siRNA *TSPAN14* NCI-H460 cells. Cell nuclei are labeled with Hoechst 33342 (blue). The scale bar in the pictures marks a length of 100 μm. The histogram shows the Tspan14 expression normalized to the control. All results are from at least three independent experiments. *p* < 0.05 (*), *p* < 0.001 (***) and *p* < 0.0001 (****) indicate statistically significant difference between siRNA NC and siRNA *TSPAN14* transfected cells relative to control, while *p* < 0.001 (###) and *p* < 0.0001 (####) indicates statistically significant difference between siRNA NC and siRNA *TSPAN14*.

To investigate the role of Tspan14 in the invasive characteristics of NSCLC cell lines, we used siRNA transfection to suppress Tspan14 expression. Having the highest Tspan14 expression among the examined NSCLC cell lines, NCI-H460 cells were chosen for transfection. After transfection with siRNA *TSPAN14*, *TSPAN14* gene expression in NCI-H460 cells was significantly decreased compared with the control (3.6-fold, *p* = 0.0001), as well as a non-coding siRNA-negative control (siRNA NC, 2.9-fold, *p* = 0.0001, Figure 3a). Additionally, we compared the expression of Tspan14 at the protein level. The results shown in Figure 3b demonstrate that the Tspan14 protein expression in transfected NCI-H460 cells was also reduced compared with the control (1.8-fold, *p* = 0.0052) and siRNA NC (1.7-fold, *p* = 0.0033).

**Figure 4:**
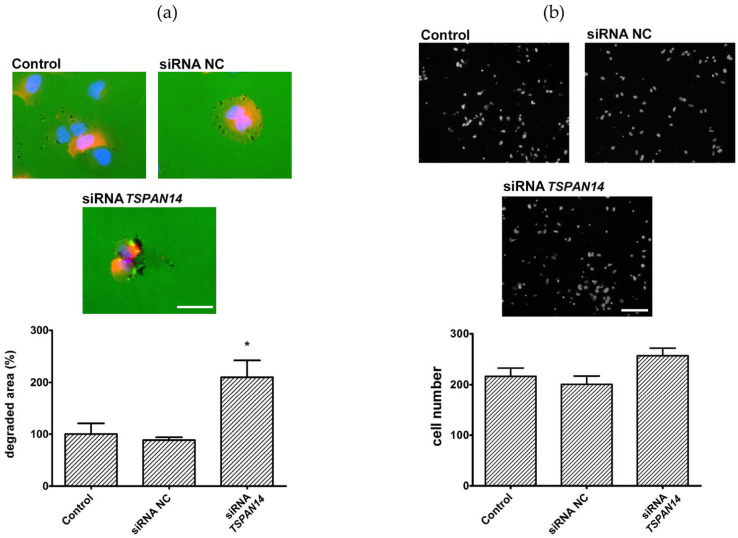
NCI-H460 cells with silenced *TSPAN14* expression demonstrate higher migratory and invasive potential. (**a**) Representative images of gelatin degradation by control, siRNA NC, and siRNA *TSPAN14* transfected cells, attributed to histograms, show a degraded surface of gelatin under the cell per total surface of the cell. The scale bar in the figures marks a length of 50 μm. (**b**) Representative images of control, siRNA NC, and siRNA *TSPAN14* cells that migrated through Matrigel^®^ matrix to the other side of the porous membrane; the histograms represent an average number of cells per field, in 10 independent fields per membrane, from three independent experiments. The scale bar in the pictures marks a length of 100 μm. All results are from at least three independent experiments. *p* < 0.05 (*) indicates a statistically significant difference between siRNA *TSPAN14* transfected cells and control cells.

Further, it was analyzed whether silenced Tspan14 expression in NCI-H460 cells decreases the invasive potential of these cells. As shown in Figure 4a, NCI-H460 cells transfected with siRNA *TSPAN14* have a higher ability to degrade gelatin compared to control (2.1-fold, *p* = 0.02) and siRNA-NC groups (2.3-fold, *p* = 0.01). No statistically significant difference between the groups was observed in the cell invasion assay when comparing the number of cells degrading the matrix and passing through the membrane (Figure 4b).

**Figure 5:**
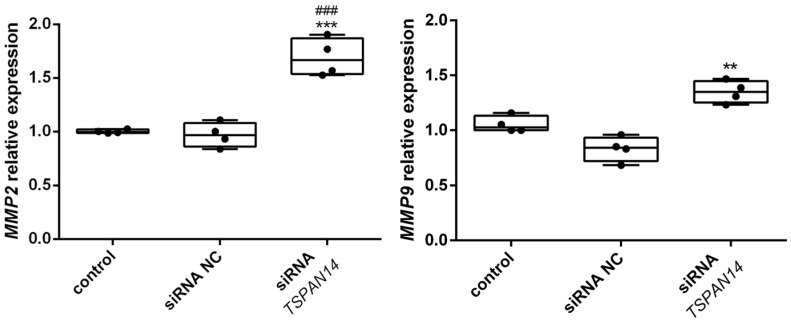
The qPCR analysis of matrix metalloproteinases *MMP2* and *MMP9* gene expression in control, the siRNA NC, and siRNA *TSPAN14* transfected NCI-H460 cells. The gene expression of *MMP2* and *MMP9* was normalized to *ACTB* as endogenous control. All results are from four independent experiments. *p* < 0.01 (**) and *p* < 0.001 (***) indicate statistically significant differences between siRNA *TSPAN14* transfected cells and control cells, while *p* < 0.001 (###) indicates a statistically significant difference between siRNA NC and siRNA *TSPAN14* transfected cells.

Additionally, the expression of the MMP genes was analyzed (*MMP2* and *MMP9*) in non-transfected control and *TSPAN14*-silenced NCI-H460 cells (Figure 5). We found that *MMP2* (1.7-fold, *p* = 0.0041) and *MMP9* (1.4-fold, *p* = 0.009) were significantly increased in NCI-H460 *TSPAN14*-silenced cells compared with non-transfected control.

## 4. Discussion

This study investigated the role of Tspan14 in NSCLC cells. To the best of our knowledge, the role of Tspan14 has not been investigated in NSCLC cells so far. Our analysis showed that the *TSPAN14* gene expression level was decreased in tumor tissue derived from NSCLC patient samples. On top of that, *TSPAN14* decreased expression correlated with pleural invasion patients. Pleural invasion, defined as cancer cell penetration beyond the elastic layer of lung visceral pleura, has been considered a factor in patients’ poor prognosis, and an indicator of NSCLC invasiveness and aggressiveness [22,23]. Ultimately, decreased expression of *TSPAN14* indicated a low survival rate, compared to other NSCLC patients.

Tspans, the transmembrane proteins, act as platforms for organizing and connecting cell-surface proteins, assisting in cell signalization and determination of cell fate [7]. In previous studies, it has been confirmed that proteins from the Tspan family, depending on the context, promote or suppress the progression of different tumors. [5,6,24]. CD9 (Tspan4) and CD81 (Tspan28), depending on tumor type, can promote or suppress disease progression [25,26,27,28], while some other Tspan proteins, such as CD82 (Tspan27) and CD63 (Tspan30), have solely been established as tumor suppressors [29,30,31,32]. In NSCLC, a decreased expression of CD82 [33] and CD63 [34] and an elevated expression in CD151 (Tspan24) [35] are negative prognostic factors.

Although our patients’ cohort was small and heterogonous, it allowed us to hypothesize that a decreased *TSPAN14* expression could be a candidate indicating invasiveness and poor prognosis of NSCLC. Our hypothesis was reinforced with in silico analyses of OS and PFS that were also performed on heterogeneous cohorts of 1144 and 591 NSCLC patients, respectively.

Further analysis of the Tspan14 expression profile in different NSCLC cell lines confirmed decreased expression in cancer cells with higher invasive potential. Our results showed that NSCLC cancer cells with the foremost potential to degrade the gelatin/matrix and migrate toward the molecular attractants had low expression of Tspan14 as well. Correspondingly, NSCLC cancer cells with the highest expression of Tspan14 had a low potential to invade and migrate through the artificial ECM models. What’s more, silencing the *TSPAN14* in these cancer cells increased their ability to degrade gelatin, and to a limited extent, increased the migratory potential. Similar to our results, previous studies have shown an association of other proteins from the Tspans family with cell motility and invasive abilities of breast tumor cells. Specifically, the α3β-integrin-tetraspanin protein complex has been linked to an invasive phenotype of the MDA-MB-231 breast cancer cell line, via the activation of MMP-2 and signaling pathways controlling the cytoskeleton [36]. CD9, CD81, CD82, and CD151, directly or through protein kinase C, interact with integrins, activating integrin-dependent cell motility [36,37].

Invasiveness and metastasis are the main features of malignant tumors, while MMPs are essential in metastasis initiation, clearing the surrounding ECM for the cancer cells to move away from the primary tumor. *MMP2* and *MMP9* expression in metastatic cancer cells is often elevated and they are considered of particular importance in developing metastatic potential [38], particularly in tissues where ECM is abundant in collagen type IV. Our results showed that silencing of *TSPAN14* in NCI-H460 cells was followed by an increase in *MMP2* and *MMP9*, gelatinases responsible for basement matrix collagen type IV degradation. This increased expression of *MMP2* and *MMP9* in NCI-H460 *TSPAN14*-silenced cells is in coherence with the results obtained in the gelatin degradation assay. The lack of significant difference between *TSPAN14*-silenced and control cells obtained in the Matrigel^®^ invasion assay implies that *TSPAN14* is more involved in extracellular matrix degradation than in the migratory capacity of the cell.

Elevated MMP-2 and MMP-9 protein expression can be induced by activated Notch-1 [39]. As previously mentioned, the TspanC8 subfamily of proteins has been deemed a regulator of ADAM10, which in turn controls Notch activation and signalization [14,15]. It has been proposed that both Tspan12 and Tspan14 are regulators of bone morphogenic protein (BMP) signalization—governor of many developmental and homeostatic processes, through ADAM10 activation [40]. In addition, Tspan14 has been confirmed as an interacting protein and a promoter of ADAM10 maturation in the HEK-293T cell line [15]. Considering that, we speculate that Tspan14 affects the migration and invasion of NSCLC cells by regulating the expression of *MMP2* and *MMP9*, through the regulation of ADAM10 and Notch-1 proteins.

Earlier, we revealed that mutational inactivation in *TSPAN14* could play a role in NSCLC promotion and probably explain the shorter survival of patients bearing this mutation [13]. However, decreased expression of *TSPAN14* could be a result of miRNA silencing in metastatic cells [41]. Here, the association of decreased *TSPAN14* expression with the NSCLC invasive potential and poor patients’ survival was demonstrated for the first time. Our findings are supported by the in silico analyses of OS and PFS in NSCLC patients with low and high *TSPAN14* expression. Therefore, our data provide critical insight into the role of *TSPAN14* in the pathogenesis of NSCLC, particularly when associated with processes crucial in tumor metastasis. We are aware of the study limitations: (i) a low number of NSCLC patients; (ii) the necessity to access the function of Tspan14; (iii) the necessity to provide evidence for a negative correlation between Tspan14 expression and NSCLC invasion in patient-derived cells. With a precise mechanism yet to be discovered, we propose that Tspan14 in NSCLC cells acts as a negative regulator of *MMP2* and *MMP9* expression. Most importantly, this study brought to attention that the expression profile of *TSPAN14* could be used as one of the prognostic indicators of metastasis development in NSCLC patients.

## Figures and Tables

**Table 1:** Clinicopathological parameters of the 40 NSCLC patients.

Parameter	Total NP **	Decreased *TSPAN14* Expression		*p*-Value
		Yes (%)	No (%)	
Total	40	14 (35.0)	26 (65.0)	
NSCLC subtype				
Adenocarcinoma	16	6 (37.5)	10 (62.5)	1
Squamous cell carcinoma	24	8 (33.3)	16 (66.6)	
Histological grade *				
g1	9	4 (44.4)	5 (55.4)	0.79
g2	26	9 (34.6)	17 (65.4)	
g3	5	1 (20.0)	4 (80.0)	
Stage				
I	2	1 (50.0)	1 (50.0)	1
II	17	6 (35.3)	11 (64.7)	
III	21	7 (33.3)	14 (66.6)	
Lymph node invasion				
Negative	8	3 (37.5)	5 (62.5)	1
Positive	32	11 (34.3)	21 (65.7)	
Pleural invasion				
Negative	31	8 (25.8)	23 (74.2)	0.044
Positive	9	6 (66.6)	3 (33.3)	

* g1, well-differentiated; g2, moderately differentiated; g3, poorly differentiated. ** NP, number of patients per group.

## Data Availability

Not applicable.

## Supplementary Materials

The following supporting information can be downloaded at: https://www.mdpi.com/article/10.3390/life12091291/s1, Figure S1: Univariable and multivariable Cox proportional hazards analyses for overall survival (OS) presented by forest plot.
