# Supplementary figures and images for: Decreased TSPAN14 Expression Contributes to NSCLC Progression
Source: Life (Basel). 2022 Aug 23;12(9):1291. doi: 10.3390/life12091291 (PMC9506201; doi:10.3390/life12091291)

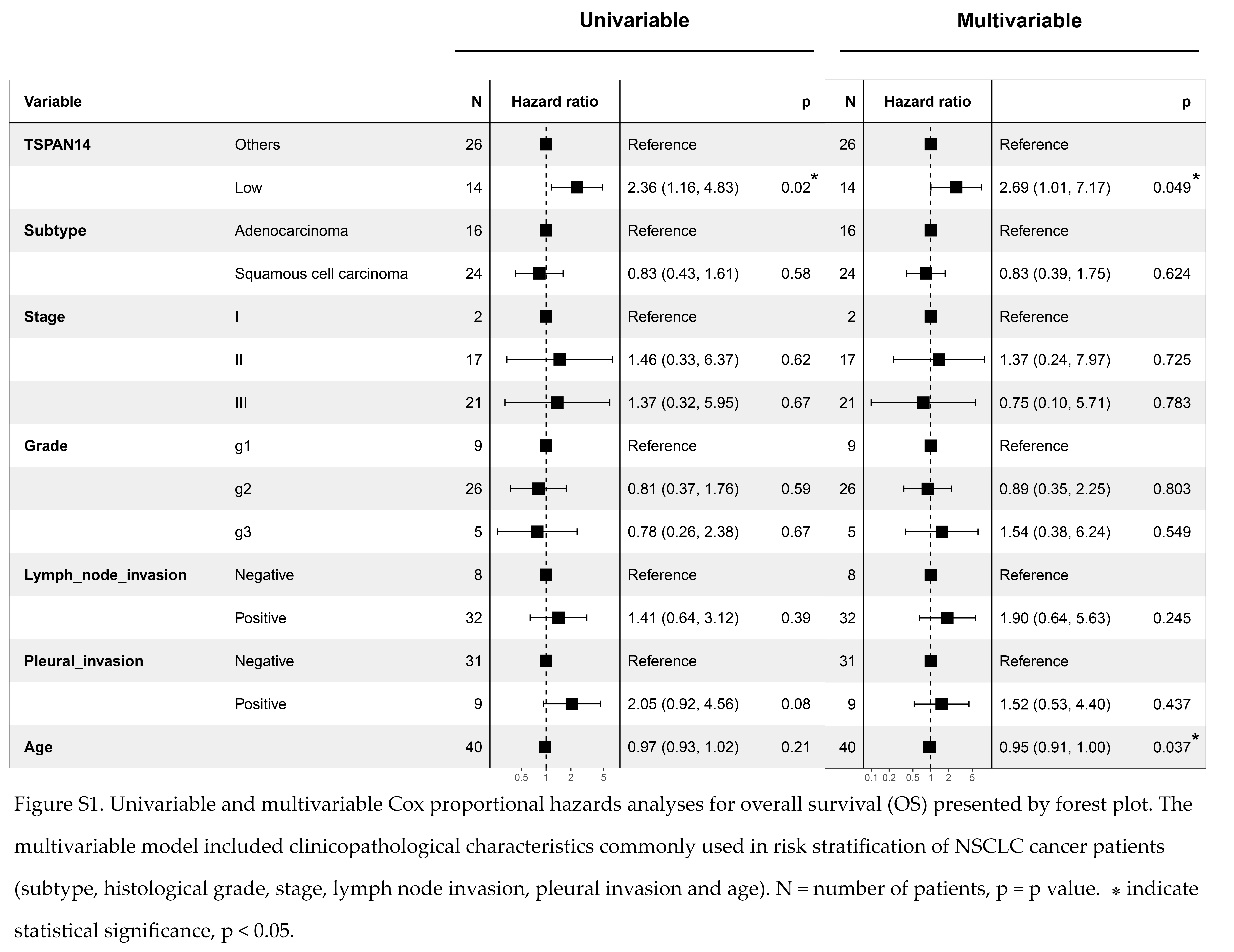

Supplement: Supplementary file 1 [file life-12-01291-s001.zip › life-1849082-supplementary.tif]
